# Genome-resolved metagenomics reveals rare taxa and functional traits in two contrasting agricultural fields

**DOI:** 10.1093/ismeco/ycag268

**Published:** 2026-09-21

**Authors:** Ioanna Chatzigiannidou, Tanvi Taparia, Pi Lærke Johansen, Lelde Berzina, Joseph Nesme, Morten Petersen, Søren J Sørensen, Susanne Brix, Karsten Kristiansen

**Affiliations:** Department of Biotechnology and Biomedicine, Technical University of Denmark, Kongens Lyngby 2800, Denmark; Department of Biology, University of Copenhagen, Copenhagen 2100, Denmark; Department of Biotechnology and Biomedicine, Technical University of Denmark, Kongens Lyngby 2800, Denmark; Department of Biology, University of Copenhagen, Copenhagen 2100, Denmark; Department of Biology, University of Copenhagen, Copenhagen 2100, Denmark; Department of Plant and Environmental Sciences, University of Copenhagen, Frederiksberg 1871, Denmark; Department of Biology, University of Copenhagen, Copenhagen 2100, Denmark; Department of Biotechnology and Biomedicine, Technical University of Denmark, Kongens Lyngby 2800, Denmark; Department of Biology, University of Copenhagen, Copenhagen 2100, Denmark

**Keywords:** ultra-deep sequencing, microbial dark matter, functional metagenomics, nitrogen cycling

## Abstract

Agricultural soils harbor highly diverse and functionally rich microbial communities, yet much of their genomic potential remains unexplored. Here, we present a genome-resolved metagenomic analysis of 455 soil samples collected at different depths and seasonal stages across two Danish agricultural fields representing sandy and clay soil types. Ultra-deep shotgun sequencing enabled the recovery of 58 831 medium to high-quality prokaryotic metagenome-assembled genomes (MAGs), which were dereplicated into 56 340 strain-level MAGs and 9090 species-level genome bins (SGBs) spanning 49 prokaryotic phyla. The majority of the SGBs represented previously uncharacterized species. Functional annotation revealed extensive biosynthetic and metabolic potential, including 46 913 biosynthetic gene clusters and prevalent genes linked to nitrogen cycling. Notably, 378 MAGs harbored the nitrous oxide reductase gene *nosZ*. Soil type and depth strongly influenced microbial diversity and functional traits. These findings provide a resource for linking microbial traits to soil function of potential value for better sustainable agricultural practice.

## Introduction

Soil is a fundamental component of terrestrial ecosystems, mediating essential processes such as nutrient cycling and carbon sequestration. Central to these functions is the soil microbiome, encompassing complex and dynamic communities of bacteria, fungi, archaea, and other microorganisms that drive biogeochemical transformations, enhance nutrient availability, and support plant health through symbiotic and mutualistic interactions [1, 2]. In agricultural systems, these microbial communities directly influence crop yields and resilience to environmental stressors, underscoring the pivotal role of the microbiome in sustaining both ecological balance and food security. Thus, deep understanding of the soil microbiome composition and functionality is essential to support plant productivity and predict responses to environmental changes [3, 4].

Despite its importance, comprehensive characterization of soil microbial communities remains challenging. Traditional culture-based methods capture only a small fraction of the microbial diversity [5], leaving much of the soil microbiome unexplored. Metagenomics, as a culture-independent approach, has significantly advanced our understanding of microbial diversity and function in soil ecosystems [6, 7]. However, agricultural soils pose particular challenges for metagenomic analysis due to their exceptionally high microbial complexity and diversity [8]. A single gram of soil may contain thousands to millions of microbial species [9], many of which are rare or low in abundance. Moreover, soil microbial communities are highly heterogeneous across spatial scales [10], further complicating efforts to recover representative genomes.

To address these challenges, high sequencing depth is increasingly recognized as essential for capturing the full extent of microbial diversity and facilitating genome reconstruction of rare or low abundance taxa [11–13]. In parallel, optimized assembly strategies are critical for improving the recovery of complete genes and genomes from complex soil metagenomes. Thus recently, different groups have exploited shotgun and long-reads metagenomics data for characterizing agricultural and crop-associated soil bacteria [14–17], yet a substantial portion of the microbial phylogenetic and functional diversity remains uncharacterized. This is particularly evident in agricultural soils, where genome recovery has proven more difficult compared to other soil and sediment environments [16] due to the higher microdiversity and the lack of dominant species in this environment.

Here, we used ultra-deep shotgun metagenomic sequencing of 455 agricultural soil samples to test the hypothesis that extensive sequencing depth combined with an optimized assembly strategy [18] would substantially improve genome recovery from low-abundance microorganisms that are typically underrepresented in agricultural soil genome catalogues. To maximize recovery of microbial diversity, we included samples from two fields with contrasting soil properties (clay and sandy soils), collected at two depths and across six time points throughout the growing season, thereby capturing both spatial and temporal variation in community composition. We hypothesized that this broad sampling design would increase detection of rare and habitat-specific taxa and enable reconstruction of a more comprehensive representation of the agricultural soil microbiome. By compiling this expanded prokaryotic genome catalogue, we aimed to improve the understanding of microbial diversity in agricultural soils and to provide a more complete assessment of the genetic potential encoded by these communities.

## Materials and methods

### Sample collection

Soil samples were collected from two different agricultural potato fields in Northern Denmark (GPS coordinates sandy soil: 57.149500, 10.233722; GPS coordinates clay soil: 57.195949, 9.79221) at two different depths (5–15 cm and 30–40 cm) (Table 1), and at six time points across the growth season of potato crops: (i) at seeding, (ii) plant emergence, (iii) plant elongation, (iv) plant flowering, (v) tuber growth, and (vi) harvest. For each tested condition, samples were collected from five biological replicate sites within four sampling areas in each field, with a minimum distance of five meters between sites. Due to logistical and practical constraints, few sample points were missing. Twenty-five grams of soil were sifted through a sieve, maximum diameter of 2 mm, to eliminate larger particles. The sample was then transferred to a 15 ml tube with 20% glycerol in PBS. After thorough mixing, an aliquot corresponding to 250 mg of soil was immediately transferred to an extraction tube, and this tube and the 15 ml tube were stored at −80°C until further use.

**Table 1:** Physiological profile of the two fields.

Soil type	Depth (cm)	Location (GPS coordinates)	Nitrate (μg/L)	Phosphate (μg/L)	Ammonium (μg/L)
Sandy	5–15	57.149500, 10.233722	1865.12	2191.37	39.04
Sandy	30–40	57.149500, 10.233722	408.23	2170.59	444.12
Clay	5–15	57.195949, 9.79221	2334.54	2278.57	44.00
Clay	30–40	57.195949, 9.79221	1908.57	2270.32	228.61

### Soil chemical characterization

For determination of phosphate, ammonium and nitrate content, 10 g of fresh soil was shaken at 200 rpm in 50 ml of double distilled water for 30 min and the filtered extracts were measured spectrophotometrically in a SEAL AA500 continuous flow analyzer (Seal Analytical, Mequon, USA) in technical replicates. Determination was performed according to standard protocols [19]. Thus, orthophosphate concentrations were measured with method A-005-19 Rev.1 using the Murphy and Riley reaction of ortho-phosphate with molybdate and antimony, followed by reduction with ascorbic acid at a pH < 1 and colorimetric measurements at 880 nm [20]. Ammonium determination was performed using method A-043-19 Rev. 0 based on the Berthelot reaction, with sodium nitroprusside as a catalyst and colorimetric measurements at 660 nm [19]. Nitrate determination was done using method A-044-19 Rev. 3 whereby nitrate was reduced to nitrite at pH 7.5 in a copperized cadmium reduction coil. Finally, total nitrite was quantified using Griess reaction which involved diazotization under acidic condition leading to the formation of a reddish-purple azo dye, with colorimetric measurements at 543 nm.

### DNA extraction

Before extraction, samples were spun for 10 min at 4000 × g, room temperature. After removal of the supernatant, the 250 mg of soil sample was transferred to MN Bead Tube Type A, and DNA extraction was conducted using the NucleoSpin Soil, Mini kit (740780.250, Macherey-Nagel GmbH&Co, Germany). The sample material was resuspended in SL1 lysis buffer, supplemented with the Enhancer XS, followed by mechanical disruption on a Precellys® Tissue homogenizer (Bertin Technologies SAS, France). PCR inhibitors and proteins were precipitated with SL3 lysis buffer and pelleted by 10 min centrifugation at 11000 × g. Next, samples were loaded on NucleoSpin® Soil columns and subsequently washed with Binding Buffer SB and Wash Buffers SW1/SW2 to remove residual humic substances and inhibitors. After 10 min of silica membrane drying, samples were eluted in 80°C pre-warmed SE Elution buffer (5 mM Tris/HCl, pH 8.5) and incubated for 10 min at room temperature, finished with 2 min centrifugation at 11000 × g. Samples were stored at −80°C until further use.

### Shotgun metagenomic sequencing

Libraries for metagenomic shotgun sequencing were constructed using MGIEasy FS DNA Library Prep set (1 000 017 572, MGI Tech, China). In brief library preparation included DNA shearing for 8 min at 30°C and magnetic bead-based size selection, end repair and adapter ligation followed by magnetic bead cleanup, PCR, and magnetic bead-based size selection of the final product. Final library concentration and fragment size were determined by Qubit 1× dsDNA HS Assay Kit (Q33231, ThermoFisher, USA) and High Sensitivity D1000 Assay Kit (5067-5584, Agilent Technologies, USA), respectively. Pooled, circularized and barcoded libraries were used for DNA nanoball (DNB) formation and further analyzed on DNBSEQ-T10x4RS (MGI Tech, China) using DNBSEQ-T10 × 4RS High-throughput Sequencing Set (FCL PE150) (940-000100-00, MGI Tech, China) according to manufacturer’s instructions.

### Sequence processing and assembly

Due to a few missing samples and samples that did not yield a sufficient number of reads during sequencing, the final dataset consisted of 455 sequenced samples. Quality control of the raw reads and adapter removal were performed with Trimgalore (v 0.6.4) with the following parameters “--length 50 --stringency 5 --clip_R1 15 --clip_R2 15”. The above resulted in ~60 Tb of clean sequencing reads, varying from 50.4 Gb to 588.4 Gb per sample with a median of 110 Gb per sample [18]. Sequencing coverage per sample was estimated with nonpareil (v 3.4.1) [11] using the kmer option. Biological replicates (n = 5) belonging to the same field, depth, and time point were co-assembled, resulting in 96 assemblies. Co-assembly was chosen over individual sample assembly because it has been shown to improve assembly quality and increase genome recovery, particularly in highly diverse soil metagenomes [18]. Co-assembly of a limited number of samples was selected instead of co-assembly of all samples due to prohibitive computational cost that the latter would require. Moreover, we aimed to reduce the inherited limitations of co-assembly artefacts, i.e. chimeric assemblies, by only combining biological replicates that would be expected to host similar strains. The co-assemblies were generated using MEGAHIT (v 1.2.9) [21] with the following kmer parameters “--k-min 27 --k-max 137 --k-step 20”. Only contigs with a minimum size of 2000 bp were retained for further analysis. Read recruitment to assemblies was evaluated using the contig mode of coverM (v 0.6.1) [22].

### Metagenome binning and refinement

Binning was performed separately for each co-assembly using contigs ≥2 kb and coverage information derived from read mapping of the samples used for the given assembly. Reads were mapped to assemblies using Bowtie2 (v2.5.1) with default paired-end settings, and resulting BAM files were filtered, sorted, and indexed with SAMtools [23]. Genome binning was performed with VAMB (v 4.1.3) [24] using default parameters including both vae and aae models and with Semibin (v 1.5.1) [25] using default parameters in the self-supervised mode. Bins larger than 200 kb were subsequently refined using MAGpurify (v 2.1.2) [26] with default parameters based on a) tetranucleotide frequency and b) GC content. After refinement, bin quality was evaluated with CheckM2 (v 1.0.1) [27] using the general model, and bins with completeness ≥50% and contamination <10% were retained. DAS_Tool (v 1.1.6; internal quality filter set to 0) [28] was used to resolve contigs overlapping across bins produced through different binners and ensure that each contig was assigned to a single bin. The above resulted in a total of 58 831 MAGs with medium to high quality.

To remove redundancy, MAGs were dereplicated using dRep (v 3.4.5) [29] utilizing multiround primary clustering. Specifically, the function dRep dereplicate was used and the ANI threshold for secondary clustering (−sa) was set to 95% similarity for the species level (SGBs) catalogue and to 99.9% similarity for the strain level (stMAG) catalogue.

GTDB-tk (v 2.4.0) (GTDB release 220) [30] was used to assign taxonomy to bacterial and archaeal MAGs. MAGs with completeness ≥90% and contamination <5% were evaluated on whether they have SSU (16S rRNA) and LSU (5S and 23S rRNA) with infernal (v 1.1.4) [31] and the number of tRNAs per genome was evaluated with tRNAscan-SE (v 2.0.12) [32]. MAGs with a positive hit for all three ribosomal subunits and at least 18 tRNAs were considered high quality, according to MIMAG standards [33].

MAGs with completeness ≥90% and contamination <5% were considered near complete [34]. MAGs that fulfilled the medium-quality criteria, according to CheckM2, but had a total genome size larger than expected for prokaryotes, >15 Mb, were evaluated to determine if they were eukaryotic genomes with EukCC (v 2.1.0) [35]. Genomes with eukaryotic completeness >30% and prokaryotic completeness <80% were removed as eukaryotic genomes. All stMAGs that belonged to the given SGB were also removed.

### Phylogenetic tree

Phylogenetic trees were constructed separately for bacteria and archaea using iqtree (v. 2.3.5) [36] with the extended model selection -m MFP and gtdb-tk alignment files as input. The trees were visualized in iTOL [37].

### Comparison with publicly available MAGs

We compared our SGB catalogue with different genome catalogues to identify whether the MAGs belonged to a known species. As a general catalogue of bacterial and archaeal genomes the GTDB reference database (v 220) [38] (113 104 genomes) and the “Representative genomes” catalogue of progenomes3 [39] (41 777 genomes) were used. Additionally, the SGB catalogue was compared against the Spire Representative Genomes from the extensive MAG collection SPIRE [40] (107 008 species level MAGs), as well as the GEM catalogue [41] (52 515 MAGs) and a publicly available list of soil SGBs (SMAG catalogue [14], 21 077 species level MAGs). Genome comparison against each one of the databases was performed using dRep compare, using fastANI (v1.34) for ANI comparisons, multiround primary clustering at 90% ANI with a chucksize of 20 000, and greedy secondary clustering at 95% ANI. SGBs that had ≥95% ANI similarity with at least one genome from at least one database were considered as known, while SGBs with no ANI similarity ≥95% were considered as previously uncharacterized.

### Gene prediction and annotation

Open reading frames (ORFs) were predicted in contigs >2000 bp for every assembly using prodigal (v 2.6.3) [42]. To construct a unique gene catalogue, in which we removed redundancy while maintaining biologically relevant functional variation, all predicted ORFs across assemblies were combined and clustered by mmseqs linclust (Release 14-7e284) [43] on the nucleotide sequences with the following parameter “-c 0.9 --min-seq-id 0.95”, which removes redundancy and prevents fragmented genes to cluster with complete genes and “--cov-mode 1”, which allows to cluster protein fragments. “--kmer-per-seq 80” was chosen as a good trade-off between maximizing sensitivity and reduction of computational resources. Following dereplication, the unique genes were annotated by eggNOG-mapper (v 2.1.7) [44] “–itype CDS –translate -diamond.basic”. Additionally, the gene catalogue was dereplicated based on 90% amino acid sequence similarity with a minimum coverage of 80% to acquire a catalogue of unique homologous proteins. Mmseqs linclust run with the following parameters “--cov-mode 1 -c 0.8 --kmer-per-seq 80 --min-seq-id 0.90”. Finally, protein family clusters (PFCs) were acquired by dereplicating amino acid sequences based on 30% similarity according to the following parameters “--cov-mode 1 -c 0.5 --min-seq-id 0.3 --cluster-mode 2 -e 0.001” similar to [45]. The e-value cut-off was included to guarantee statistically significant similarity because of the higher number of random matches at 30% similarity and “--cluster-mode 2” to suppress fragments from becoming representative sequences. To remove spurious sequences, only PFCs harboring at least three members were considered as clusters. The 90% amino acid nonredundant protein catalogue was compared with the Global Microbial Gene Catalog v1.0 (GMGC10.90nr.faa) with mmseqs linclust (Release 14-7e284) with the following parameters “--cov-mode 1 -c 0.8 --kmer-per-seq 21 --min-seq-id 0.90”. Here, “--kmer-per-seq 21” was used to improve computational efficiency because the goal was to match against an already established protein set rather than defining cluster structure.

### Mapping genes to MAGs

Genes were assigned to the SGBs to define genome annotation. This was done by linking the contig identity of each ORF to the list of contigs that comprised each medium quality MAG. The above was used to calculate the number of ORFs per MAG. By further combining this information to the gene annotation by eggNOG-mapper, we calculated the percentage of annotated genes as the fraction of annotated ORFs over the total number of ORFs per SGB. A gene was considered annotated when it was assigned an eggNOG-OG irrespectively if the OG had a known function.

### Functional annotation of SGBs

To predict complete KEGG metabolic modules for each SGB, first ORFs per SGB were predicted with prodigal (v2.6.3) [42] using default parameters and gene functions were annotated with KofamScan (v 20 190 424) [46] using default parameters and the -f mapper option. The produced table was used as input for MetaPathPredict [47], run with default parameters and option -a kofamscan, to predict the presence of KEGG metabolic modules based on its deep learning models. AntiSMASH (v 7.0.1) [48], with default parameters and the –minimal option switched on, was used to identify Biosynthetic Gene Clusters (BGCs) for each SGB. BGCs were clustered in Gene Cluster Families (GCFs) with BiG-SLiCe (v 2.0.0) [49] and default parameters.

Genomes were considered as *nosZ* carriers based on the KofamScan results and the presence of the K00376 gene. Multiple sequence alignment was run using Clustal Omega (v1.2.1) [50] for all genes annotated as *nosZ* and 27 reference *nosZ* genes that were downloaded from NCBI (Supplementary Table 1). The tree was constructed using FastTree (v2.1.11) [51] with default parameters and was visualized using iTOL [37].

Genomes were considered to represent putative N_2_O-producer species when they (a) were predicted positive for the denitrification pathway, KEGG module M00529 according to MetaPathPredict, but did not possess the K00376 gene based on KofamScan—incomplete denitrification or (b) were positive for KEGG module M00528, ammonia → nitrite. We refined further the second category by removing *Nitrospiraceae* Palsa-1315 which has been previously classified as a member of comammox clade B [52], while retaining the *Nitrosospira* SGBs, a known Ammonia Oxidizing Bacteria (AOBs), which can also perform nitrifier denitrification [53].

### Quantification of microbial community composition

The abundance of SGBs in each sample was quantified using CoverM (v 0.6.1) [22] using the options –min-read-aligned-percent 0.75 --n 0. In per field comparisons, six samples were removed due to missing information on their field of origin, resulting in 449 samples to be considered for downstream analysis.

### Statistics

Comparisons across time points of the sample diversity or *nosZ* gene abundance were performed using a Dunn’s Test with the dunn.test() function of the dunn.test package (v1.3.7) in *R* and the FDR was controlled using the Benjamini-Hochberg adjustment for same soil type, depth, and *nosZ* clade within the same function. Comparisons of the *nosZ* gene abundance of *nosZ-*positive SGBs and putative N_2_O-producing SGBs between soil types and depth were performed with two-sided Wilcoxon test using the wilcox.test() function of the stats package (v4.5.2). Two-sided Pearson correlation between the abundance of *nosZ*-positive and putative N_2_O-producing SGBs was performed using the stat_cor() function of the ggpubr package (v.0.6.3).

## Results

### Expanding the bacterial and archaeal diversity in agricultural soil

Ultra-deep metagenomic shotgun sequencing of 455 bulk soil samples collected from two potato-growing agricultural fields was used for the de-novo reconstruction of MAGs (Fig. 1a). Even at a deep sequencing depth, sequencing coverage was estimated to vary between 30.7% and 91.5% (Supplementary Fig. 1a), while assembly read recruitment ranged from 19%–83.5%, with an average of 63.4% (Supplementary  Fig. 1b). These results underscore both the extraordinary diversity of agricultural soil microbiomes and the challenge of achieving comprehensive genome recovery in this environment, despite the application of ultra-deep sequencing and optimized assembly strategies with co-assembly of five biological replicates per sample site.

**Figure 1:**
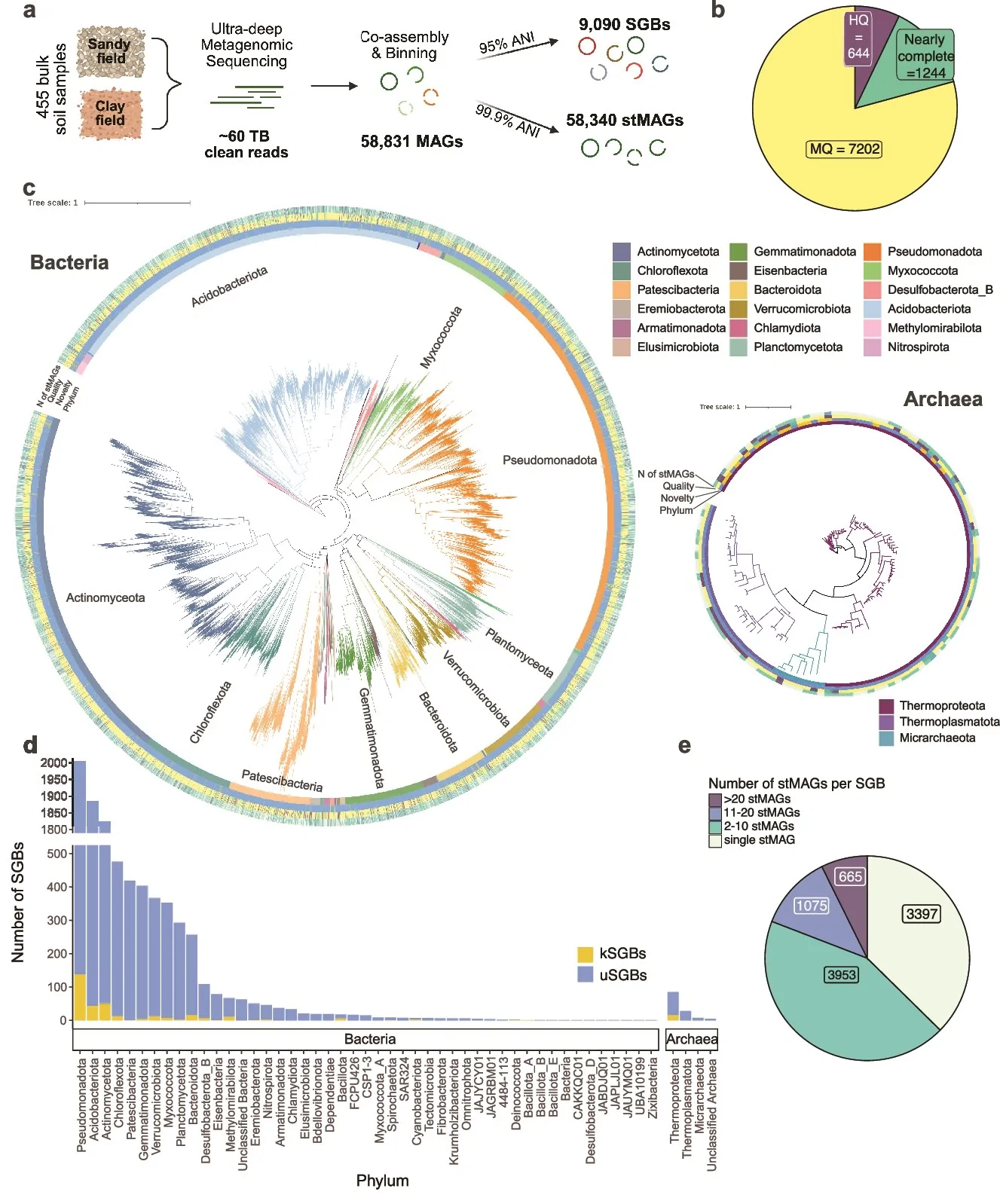
Agricultural soil harbors many uncharacterized prokaryotic species. (a) A total of 58 831 metagenome-assembled genomes (MAGs) were recovered from 455 bulk soil samples, which could be clustered into 58 340 strain-level MAGs (stMAGs) (<99.9% ANI) and 9090 species-level genome bins (SGBs) (<95% ANI), (b) quality of SGBs, (c) phylogenetic trees of the bacterial and archaeal SGBs. The color of the branches depicts the taxonomy at the phylum level. The rings (from in to out) represent (i) the taxonomy, (ii) the novelty based on similarity with reference genomes, (iii) the genome quality and (iv) the number of stMAGs corresponding to each SGB; (d) taxonomic distribution of known (kSGBs) and uncharacterized (uSGBs) genomes at the phylum level; (e) distribution of stMAGs per SGB. Parts of Figure 1a were generated with BioRender.

De-novo genome reconstruction led to the recovery of 58 831 medium to high-quality prokaryotic MAGs, according to MIMAG standards (≥50% completion and < 10% contamination) [33]. To remove redundancy across samples, MAGs were dereplicated at 99.9% ANI similarity to 56 340 strain-level MAGs (stMAGs) and 95% ANI similarity to get species-level genome bins (SGBs) comprising a total of 9090 genomes (Fig. 1a, Supplementary Fig. 1c, Supplementary  Table 2). 644 (7.1%) of the SGBs were estimated to be high quality, i.e. completion ≥90%, contamination <5%, presence of 16S, 23S and 5S rRNA genes, and at least 18 tRNA genes, and 1244 to be near complete i.e. completion ≥90%, contamination <5% [34] (Fig. 1b).

The SGBs belonged to 46 different bacterial and three different archaeal phyla, with most of them belonging to *Pseudomonadota* (2004 SGBs), *Acidobacteriota* (1884 SGBs), and *Actinomyceota* (1824 SGBs) (Fig. 1c & 1d, Supplementary Table 2). The most common archaeal phylum was *Thermoproteota* with 85 SGBs. To define the phylogenetically new SGBs, the SGBs were compared to genomes from publicly available genome catalogues (> 325 000 genomes) [14, 38–41]. We observed that 8746 SGBs, representing 96.2% of the SGBs recovered in this study, had no match using a genome similarity (ANI) ≥95% and, thus, represent previously uncharacterized species-level genomes (uSGBs) (Supplementary Fig. 1d & e, Supplementary Table 2). Therefore, our genome catalogue greatly expands the known soil diversity, adding >8.7 k new species genomes that span 47 phyla (Fig. 1d). The highest number of uSGBs belonged to the most represented phylum, *Pseudomonadota*.

Zooming in on subspecies diversity, we observed that the number of different stMAGs within each SGB varied greatly. We determined that 37.3% (3397) of the SGBs represented a sole stMAG, 43.5% (3953) of the SGBs represented 2–10 stMAGs, 11.8% (1075) of the SGBs represented 11–20 stMAGs, and 7.3% (665) of the SGBs represented more than 20 stMAGs (Fig. 1e, Supplementary Table 2). The highest number of different strain genomes (52) was recovered for an SGB belonging to the family *Myxococcaceae*. Finally, we noted that the number of retrieved stMAGs within a species depended on the prevalence of the SGBs across the dataset (Supplementary Fig. 1f).

### The uncharacterized functional and biosynthetic potential of soil prokaryotic taxa

The genetic diversity of the soil microbial catalogue was assessed by predicting and annotating genes across the assemblies, independently of whether a contig was later binned into a MAG. ORFs were predicted in each co-assembly and were further dereplicated at 99% nucleic acid identity to acquire a non-redundant list of unique genes for this dataset (Supplementary Fig. 2a), resulting in ~321 million genes. Unique genes could be further clustered into ~99 million groups based on 90% amino acid sequence similarity that represent unique homologous proteins. Finally, unique genes were clustered in larger PFCs, with 30% sequence identity, resulting in ~18 million PFCs.

The functional potential of the soil microbiome was estimated by mapping the list of unique genes against the EggNOG database. 31.2% of the genes were assigned to an EggNOG Orthologous Group (EggNOG-OG), 28.9% had a COG annotation, 28% a PFAM and 15.3% a KEGG orthology annotation (Supplementary Fig. 2b). For genes with a COG annotation, most fell within the category of amino acid transport and metabolism (Supplementary Fig. 2c).

The level of genome annotation, calculated as the percentage of genes with a match in the EggNOG database, varied greatly among SGBs ranging from 15.2% to 98.7% annotated ORFs, with an average of 87% (Fig. 2, Supplementary Table 2). The percentage of genome annotation did not depend on the genome size or the total number of predicted genes in a genome (Supplementary Fig. 3a & 3b). We observed a strong taxonomy dependent effect on the percentage of genome annotation, with genomes belonging to *Pseudomonadota*, *Spirochaetota*, *Bacillota,* and *Bacteroidota* being among the best annotated, while genomes belonging to *Patescibacteria* and *Dependentiae* were among the least annotated (Fig. 2). Unclassified bacterial and archaeal genomes had the lowest levels of gene annotation. Our results corroborate previous studies that reported the low percentage of gene annotation of *Patescibacteria* genomes [54]. *Dependentiae* and *Patescibacteria* were until recently categorized as candidate phyla with very few existing isolates and are characterized by an obligate intracellular lifestyle or epibiotic lifestyle, respectively [55, 56].

**Figure 2:**
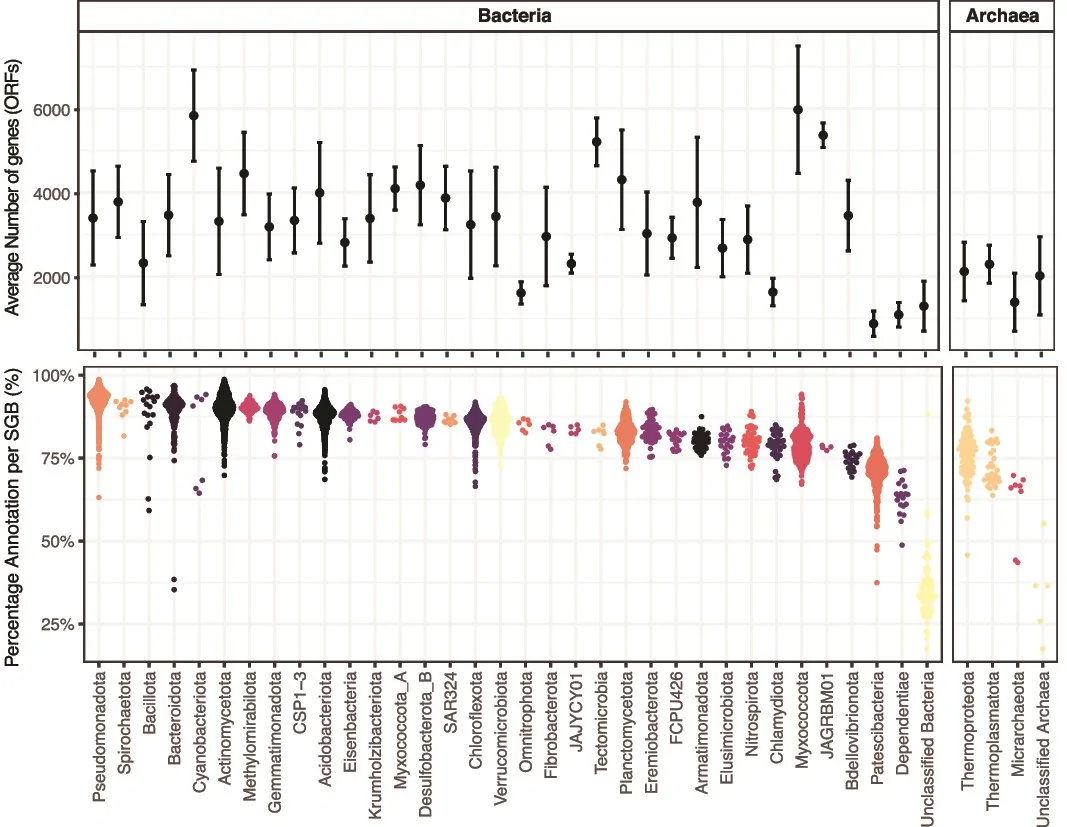
Gene annotation varies between agricultural soil microbial genomes in a phylogeny-dependent manne**r.** The first panel displays the average number and standard deviation of ORFs per phylum. The second panel shows the percentage of these ORFs annotated based on EggNOG. Each dot describes one genome.

To determine the potential of the SGBs to perform specific functions, the presence of KEGG modules in each genome was assessed. 194 different KEGG modules were searched against the SGB catalogue, and 166 of these modules were found in at least one SGB (Supplementary  Table 3). The most common modules across phyla were, not surprisingly, the ones linked to central carbohydrate metabolism (Fig. 3). Modules linked to nitrogen, methane, and sulfur metabolism were less common and only present in a few phyla. Sixteen SGBs, all classified as *Patescibacteria* were negative for all 194 KEGG modules tested in this study. Overall, *Patescibacteria* and *Dependentiae* were the phyla with the least assigned modules with an average of 2.7 (± 2.7) and 3.3 (± 1.6) identified KEGG modules per SGB, respectively (Supplementary Fig. 4, Supplementary Table 3). These low numbers of assigned KEGG modules reflect the adaptation to the obligate intracellular lifestyle for *Dependentiae* and obligate epibiont nature of *Patescibacteria*, allowing for the evolutionary reduction in genes involved in otherwise essential metabolic pathways [55, 56]. At the species level, we discovered three SGBs harboring the methane oxidation pathway (methanotrophs), belonging to the genus *Methylocella* (n = 2) and *Methylocystis* (n = 1) from the *Pseudomonadota* phylum (Fig. 3, Supplementary Table 3), all of which are so far uncharacterized species. Interestingly, seven different strains were recovered for one of the *Methylocella* species.

**Figure 3:**
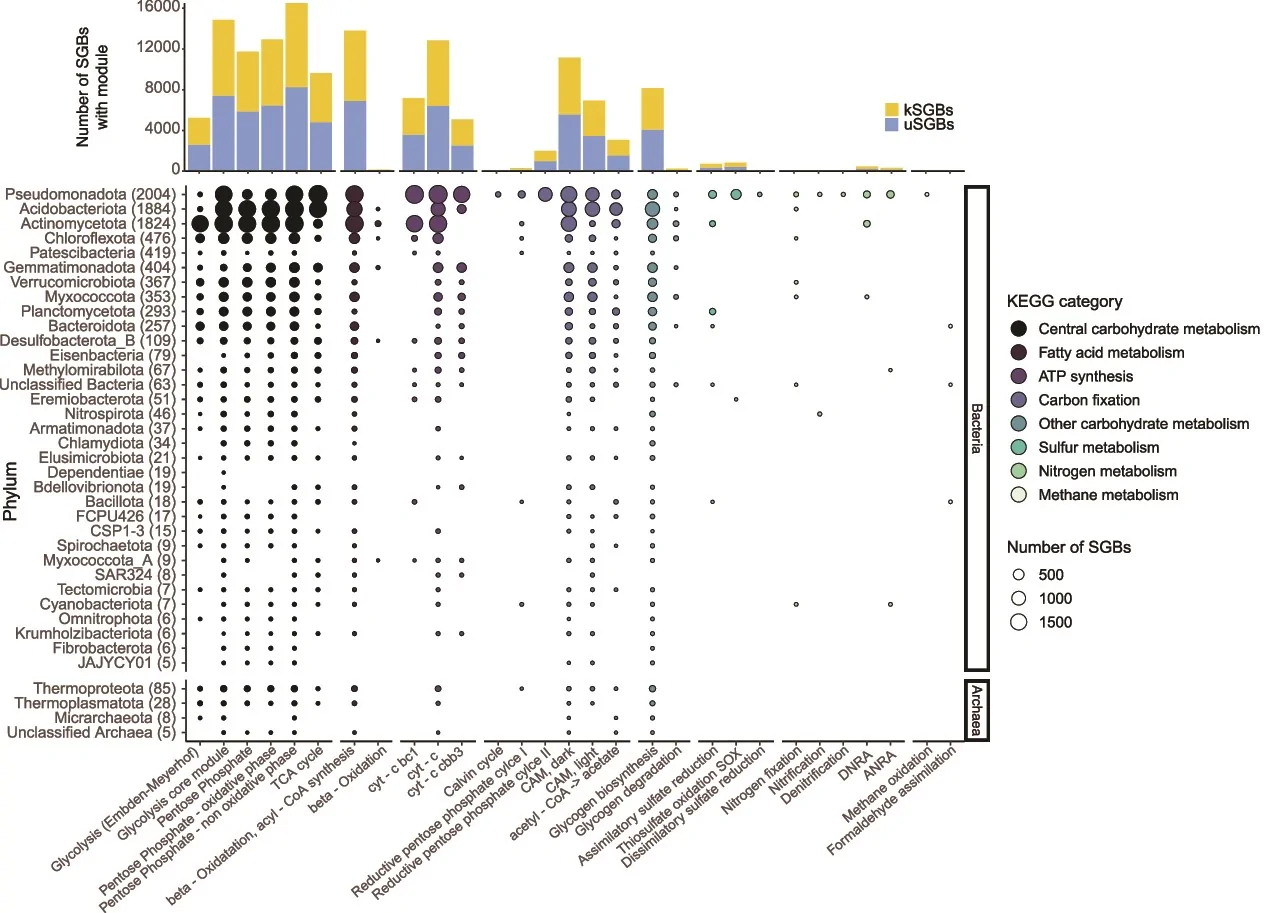
Functional diversity of agricultural prokaryotic genomes. Presence of KEGG modules involved in key metabolic pathways—Carbon, sulfur, nitrogen, and methane cycles—across phyla. The bar plot depicts the total number of SGBs across phyla within the given module, split in known and uncharacterized genomes. The size of the dot represents the number of SGBs within the given module per phylum. Only phyla with at least five SGBs are displayed.

Next, we sought to explore the biosynthetic diversity of the SGB catalogue. BGCs were predicted for each SGB with antiSMASH, resulting in 46 913 putative BGCs across the dataset, which were further clustered into 28 108 GCFs. We further grouped 61 different BGCs into categories with the most common being non-ribosomal peptide synthetases (NRPSs) (15 531 BGCs/ 7718 GCFs) and ribosomally synthesized and post-translationally modified peptides (RiPPs) (12 829 BGCs/ 6654 GCFs) (Fig. 4a, Supplementary Table 4), recapitulating previous analyses of microorganisms characterized in agricultural soil samples [14, 57]. The majority of the BGCs (92.5%) were potentially incomplete as they extended to the contig edges (Supplementary Table 4), similar to what has been reported in previous studies on environmental microbes [41]. Despite the limitations of detecting large gene clusters in short contigs, some large BGCs were found with 394 (0.8%) clusters being larger than 50 kb and 3856 (8.2%) larger than 30 kb. The largest BGC (236 kb) was detected in an SGB classified as *Amycolatopsis* representing a hybrid of thiopeptide/T1PKS/NRPS-like/indole/terpene generating modules. Across phyla, we detected the highest number of GCFs in *Acidobacteriota*, followed by *Actinomyceota*, *Pseudomonadota*, and *Myxococcota* (Fig. 4b). These findings are in contrast to the SMAG catalogue where the highest number of BGCs derived from *Pseudomonadota* genomes [14], which may be explained by the different number of genomes per phylum in the two studies. The number of unique potential gene clusters was not proportional to the number of SGBs per phylum, but some phyla were enriched for BGCs, such as *Acidobacteriota* and *Myxococcota*. *Acidobacteriota* is a phylum with known high biosynthetic potential [57–59], whereas *Myxococcota* only recently received attention for their richness in genes involved in secondary-metabolism [60]. The distribution of products varied across phyla; e.g. in *Pseudomonadota*, RiPPs were more common, whereas *Acidobacteriota* harbored more clusters encoding NRPS (Fig. 4b).

**Figure 4:**
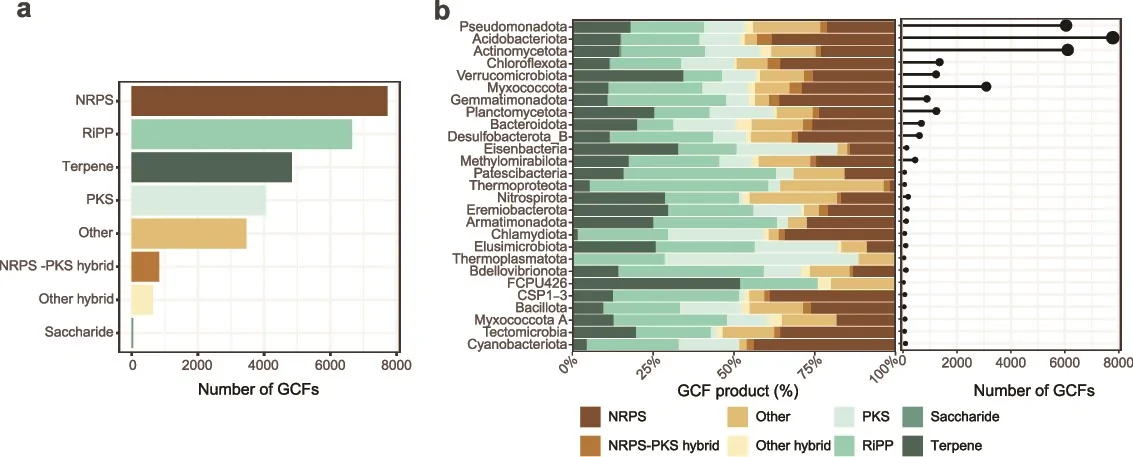
Biosynthetic potential of microorganisms in agricultural soil. (a) the number of GCFs per BGC category depicted across all SGBs, (b) phylogenetic distribution of the GCFs with the relative abundance per category and the total number of GCFs per phylum.

Most SGBs (8182, 90%) harbored at least one BGC, with BGC-richness varying from 1 to 153 BGCs and 1–129 GCFs (Supplementary Fig. 5a & 5b). The SGB with the highest number of BGCs was an uncharacterized species that belongs to the family of *Roseiflexaceae* harboring 153 BGCs, followed by a UBA5704 with 97 BGCs, and an *Amycolatopsis sp* harboring 96 BGCs. UBA5704 genomes, from soil or other environments, have been previously described to host a large number of BGCs [14, 41, 61]. Consistent with previous reports [57], the biosynthetic clusters appeared to be taxonomically conserved, with most GCF being shared mainly among genomes of the same phylum, or even genus (Supplementary Fig. 5c & d).

### Differences between different soil types and sampling depths

Next, we sought to explore whether different soil types and sampling depths were characterized by a different microbial composition and whether compositional differences reflected functional diversity especially on pathways with important ecosystem functions. Besides soil texture, the two fields and sampling depths had different nutrient profiles with higher nitrate concentrations recorded in clay soil across depths and higher ammonium concentrations in 30–40 cm samples than in 5–15 cm samples (Table 1). Nonpareil estimations based on reads diversity suggested a higher diversity in the sandy than the clay soil across time (Fig. 5b), and especially samples collected in the sandy soil at a depth of 5–15 cm had a higher diversity compared to any of the other three sample types (Supplementary Fig. 6a). High microbial diversity in sandy soil has been reported previously [62]. When considering seasonal changes, we observed that the sandy soil at depth 30–40 cm had a significantly lower diversity at the beginning of the season compared to all other time points, and the diversity of the clay soil at 5–15 cm decreased during the last two time points compared to the first two (Fig. 5b & Supplementary Table 5).

**Figure 5:**
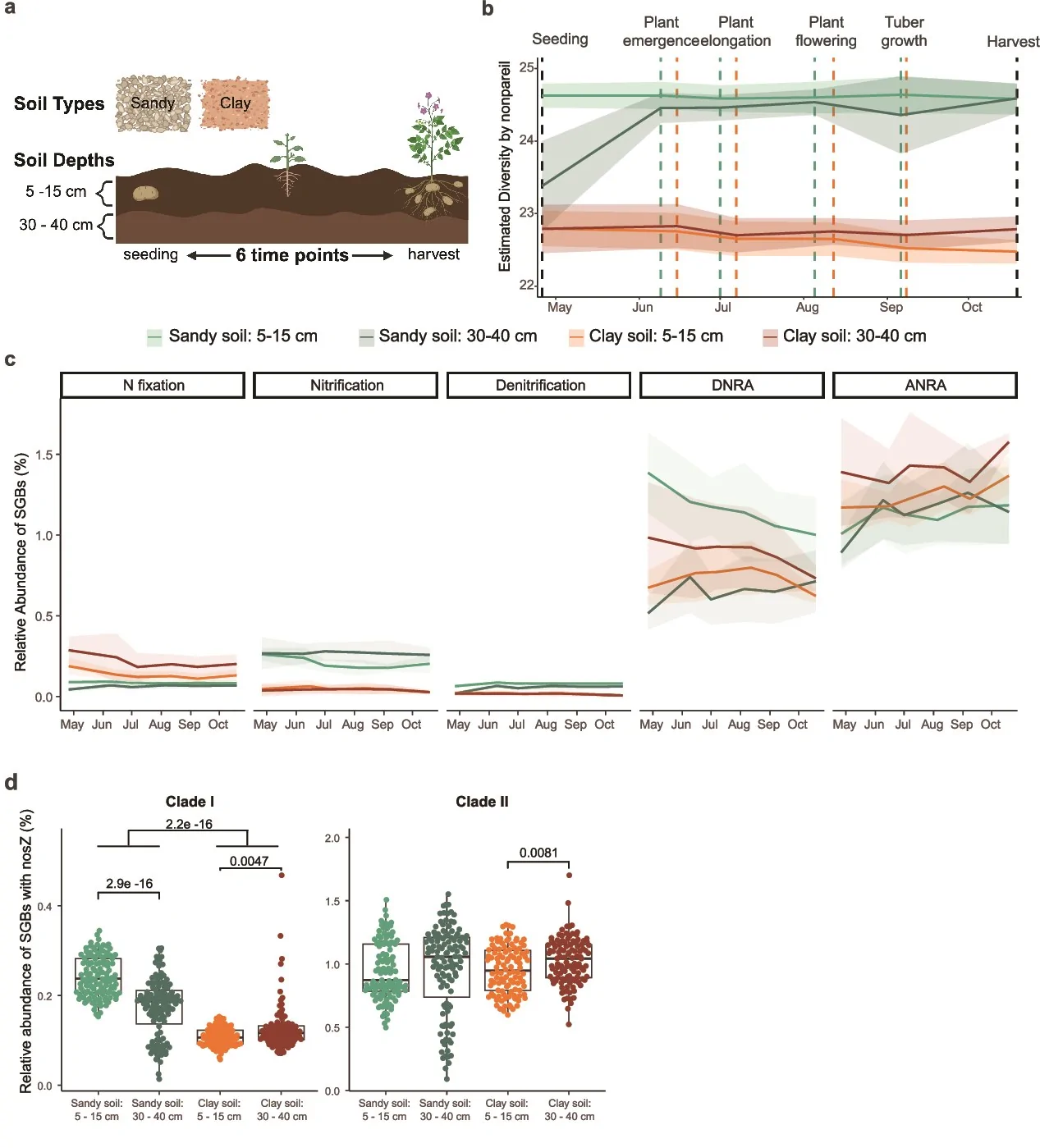
Soil microbiomes differ between soil types and depths. (a) soil samples derived from two different fields and two different depths across the growth season, (b) diversity across time estimated on read diversity—with nonpareil. The line represents the average abundance, while the ribbon reflects the standard deviation; (c) relative abundance across time of all SGBs with a given KEGG module, for modules relevant to nitrogen cycle. The line represents the average abundance, while the ribbon reflects the standard deviation; (d) relative abundance of all SGBs with a *nosZ* gene separated by *nosZ* clades. Significant differences between soil types were calculated with a two-sided Wilcoxon test. Figure 5a was generated with BioRender.

The clean reads were mapped to the SGB catalogue to investigate specific compositional and functional differences. Read recruitment to the catalogue ranged from 9.4%–57.9% (Supplementary Fig. 6b), revealing that even with a very high sequencing depth there was still a significant number of prokaryotic genomes that were not recovered. Although the recruitment rate might appear low, especially compared to the levels achieved nowadays for different environments, the values are comparable or even higher than what is commonly achieved in soil [14, 17]. A significant separation was observed between the two soil types based on the read recruitment rate (Supplementary  Fig. 6b), with the sandy soil exhibiting lower levels (*P* < .001), and the sandy samples collected at a depth of 30–40 cm to exhibit a lower mapping rate than the 5–15 cm depth samples. The comparisons between soils reflected the observed diversity differences with a strong inverse correlation between mapping rate and diversity, r2 = −0.97 and *P* < .001 (Supplementary Fig. 6c). The above highlights the difficulty in recovering genomes from the most diverse samples even at very high sequencing depths.

We attempted to examine potential functional differences between soil types and sampling depths, especially with respect to functions that may influence soil fertility or play a role in the response to a changing climate, such as pathways linked to nitrogen, methane, and sulfate cycles. When the part of the community that could be described based on the SGB catalogue was considered, we observed that the sandy soil had higher abundances of taxa harboring nitrification and denitrification pathways across the season (Fig. 5c). The total abundance of the taxa with a given N-pathway remained stable across time, except for a decrease of taxa potentially capable of dissimilatory nitrate reduction to ammonium (DNRA) in the 5–15 cm layer of sandy soil. Regarding methane metabolism, taxa harboring methane oxidation or formaldehyde assimilation pathways were more abundant in the sandy soil than the clay soil (Supplementary Fig. 6d).

DNRA retains nitrogen in soil making it available for the plants, with denitrification being the main route leading to nitrogen loss. Incomplete denitrification leads to the production of nitrous oxide (N_2_O), a major greenhouse gas [63]. Nevertheless, some microorganisms can reduce N_2_O to nitrogen thus reversing the detrimental effect of N_2_O. Therefore, microorganisms that possess the nitrous oxide reductase gene (*nosZ*) are particularly important as they represent the only known environmental sink of N_2_O. We observed that six out of the eight denitrifying SGBs carry the *nosZ* gene. Moreover, we detected the *nosZ* gene in an additional 372 SGBs that were not predicted to have the denitrification pathway. Out of the total 378 SGBs with at least one *nosZ* gene, 367 (~98.7%) were yet uncharacterized (Supplementary  Fig. 7a, Supplementary Table 6). Most of these species belonged to the phylum *Gemmatimonadota* (110), followed by *Bacteroidota* (83) and *Verrucomicrobiota* (53).

We next assigned the *nosZ* genes to the two known clades I and II [64], as previous studies have indicated that the abundance and diversity of the species with a Clade II N_2_O reductase are better indicators of the N_2_O sinking capacity of soil [65]. 39 genes were assigned to Clade I and 339 to Clade II (Supplementary Fig. 7b). In accordance with previous reports [64, 66], we found the combined abundance of species with a Clade II *nosZ* gene to be higher than those with a Clade I (Fig. 5d). Differences were also observed between soil types and depths, but these were more profound for Clade I, showing the highest abundance of SGBs having *nosZ* in the sandy soil at a 5–15 cm soil depth, and the lowest abundance in the clay soil, independently on the depth. Finally, when considering the seasonal changes, we observed that the total abundance of SGBs in both clades increased from June and onwards in the sandy soil, while Clade II but not Clade I abundance decreased in the clay soil from June and onwards (Supplementary Fig. 7c, Supplementary Table 7). The above indicates that the microbial community carrying a *nosZ* gene and potentially linked to the N_2_O sinking capacity of soil is sensitive to seasonal changes and that the dynamics depend on the soil type.

When we considered the abundance of putative N_2_O-producers, we observed that putative incomplete denitrifiers were more abundant in clay soils and in the 30–40 cm soil layer, whereas the genus *Nitrosospira*, nitrifier-denitrifier, was enriched in sandy soils and in the upper soil layer (Supplementary Fig. 8a). In clay soils, the relative abundance of incomplete denitrifiers was positively correlated with both *nosZ* Clade I and Clade II carriers (Supplementary Fig. 8b). In contrast, *nosZ* Clade I carriers were positively correlated with *Nitrosospira* in sandy soils. Collectively, these analyses revealed consistent differences between soil types and depths in the abundance of taxa carrying genes associated with nitrogen cycling.

## Discussion

Soil microbial communities are fundamental drivers of biogeochemical cycling, plant productivity, and ecosystem resilience [1, 2]. Yet their genomic diversity in intensively managed agricultural systems remains under-explored. This is highlighted by a recent study which includes >8 k shallow sequenced (median depth 4.5 Gb) soil samples across Denmark and confirms the high diversity of uncharacterized species [17]. Our study presents a deeply sequenced, genome-resolved metagenomic catalogue from Danish agricultural soils, capturing microbial diversity across soil types, depths, and crop growth stages. The catalogue contains a considerable number of high-quality SGBs not represented in current public databases, implying the presence of a substantial reservoir of previously uncharacterized microbial taxa with potential roles in soil ecosystem functioning. High-quality SGBs are essential for linking microbial species to ecological functions which are rarely achieved in soil metagenomics due to the complexity and sparsity of microbial communities. Thus, compared to global catalogues such as SMAG [14], GEM [41], and SPIRE [40], our dataset also provides information on rare and low-abundance taxa, which may play important roles in biogeochemical processes.

To establish links between bacterial species and ecological roles, we profiled the functional potential of the recovered genomes observing a wide range of metabolic and functional capabilities across the different bacterial taxa. The high BGC diversity recovered in our dataset points to complex microbial interactions and potential roles in plant-microbe signaling, competition, and defense, and could act as a resource for novel antimicrobial discovery [61]. Taxonomic enrichment of BGCs in *Acidobacteriota* and *Myxococcota*, rather than the commonly dominant *Pseudomonadota*, further suggests that agricultural soils may select for distinct biosynthetic strategies [57] with *Acidobacteriota* and *Myxococcota* known to produce antimicrobial compounds and signaling molecules that influence microbial community structure and plant health [58, 60]. Soil aggregates act as evolutionary hotspots by fostering spatial proximity of microbial consortia with distinct metabolic interactions [67]. This spatial compartmentalization could explain the taxonomic enrichment of BGCs in certain phyla like *Acidobacteriota* and *Myxococcota* and their potential roles in niche adaptation. The presence of large hybrid clusters (>200 kb) further suggests that horizontal gene transfer and genome plasticity may contribute to microbial adaptation in agricultural environments.

In addition, our dataset revealed pronounced differences in microbial communities reflecting not only soil texture but also depth. Sandy soils, with lower water-holding capacity and organic carbon, supported higher microbial diversity, consistent with the presence of more rare and low abundant bacterial taxa. These patterns are consistent with the ecological filtering effects of soil moisture, which shape microbial rarity and community evenness across biomes [68]. In the drier sandy soil, fragmentation of aqueous microhabitats limits dispersal and suppresses the dominance of fast-growing taxa, allowing rare species to persist and contribute to community structure diversity and potential functions.

While not among the most common abundant pathways, we identified several bacterial taxa that possess pathways involved in nitrogen, methane, and sulfur metabolism. Some of these taxa are described here for the first time. These pathways are particularly important due to their potential to drive key biogeochemical processes. Elevated ammonium levels in the deeper layers of sandy soil suggest reduced nitrification or higher nitrogen assimilation. Indeed, we observed higher abundance of DNRA-positive taxa in 30–40 cm sandy soil samples compared to the 5–15 cm from the same soil type, but no significant differences in the abundance of nitrifying taxa. Conversely, a higher concentration of nitrate, which likely promotes denitrifiers, was also apparent in our dataset when the 5–15 cm samples of the sandy fields were compared to deeper layers. These chemical gradients reinforce the link between the availability of potential metabolizable compounds/nutrients and microbial community structure. Additionally, temporal sampling across the crop growth season revealed dynamic shifts in the potential for specific microbial functions, while for others it remained constant. Early-season samples showed lower *nosZ* gene abundance in deeper sandy soils. These patterns likely reflect changes in root exudation, soil moisture, and nutrient availability associated with plant development stages [2, 69].

It is important to recognize that metagenomic data reflect the genetic potential of the community, not necessarily its realized function. The soil metaphenome [67], the actual product of expressed functions encoded in a metagenome, is shaped by microbial physiological status, environmental constraints, and interspecies interactions. Thus, while our catalogue provides a robust inventory of functional genes, multi-omics approaches are needed to determine which genes are actively expressed and translated under field conditions. Additionally, cultivation of representative taxa, especially those carrying ecologically relevant traits, will be essential for validating functional predictions and developing microbiome-based interventions for soil health. Understanding the dynamics of rare taxa, particularly in dry or low-biomass soils, will be a steppingstone for predicting microbial responses to climate change and land management [68, 70]. Such taxa may represent a deep reservoir of functional potential that is activated under specific environmental conditions, contributing to ecosystem resilience and adaptability.

In summary, we present a significantly expanded catalogue of soil microbes delineating differences between sandy and clay soil, including a description of changes in composition and functional potentials, especially related to nitrification, in different layers of soil during the growth season. We envisage that this catalogue will serve as a valuable resource to better understand microbe-plant interactions and to identify strategies to harness the potential of the soil microbiome to enable more robust and efficient crop production.

## Supplementary Material

Supplementary_material_ycag268

## Data Availability

The raw sequencing reads are available in the Bioproject PRJEB83729 and the SGB catalogue can be accessed via doi:10.11583/DTU.33321567.
