# Supplementary material for: Genome-resolved metagenomics reveals rare taxa and functional traits in two contrasting agricultural fields
Source: ISME Commun. 2026 Sep 21;6(1):ycag268. doi: 10.1093/ismeco/ycag268 (PMC13653607; doi:10.1093/ismeco/ycag268)
Supplement: Supplementary file 1 [file supplementary_material_ycag268.zip › Supplementary_Figures_1-8_ycag268.pdf]

SUPPLEMENTARY INFORMATION

Genome-resolved metagenomics reveals rare taxa and functional traits in two contrasting agricultural fields

Ioanna Chatzigiannidou[1], Tanvi Taparia[2], Pi Lærke Johansen[1], Lelde Berzina[2], Joseph Nesme[2], Morten Petersen[3], Søren J. Sørensen[2], Susanne Brix[1,*], Karsten Kristiansen[2,*]

1. Department of Biotechnology and Biomedicine, Technical University of Denmark, 2800 Kgs. Lyngby, Denmark
2. Department of Biology, University of Copenhagen, 2100 Copenhagen, Denmark
3. Department of Plant and Environmental Sciences, University of Copenhagen, 1871 Frederiksberg, Denmark

*Corresponding authors: Susanne Brix (sbrix@dtu.dk), Karsten Kristiansen (kk@bio.ku.dk)

Keywords: Ultra-deep sequencing, microbial dark matter, functional metagenomics, nitrogen cycling

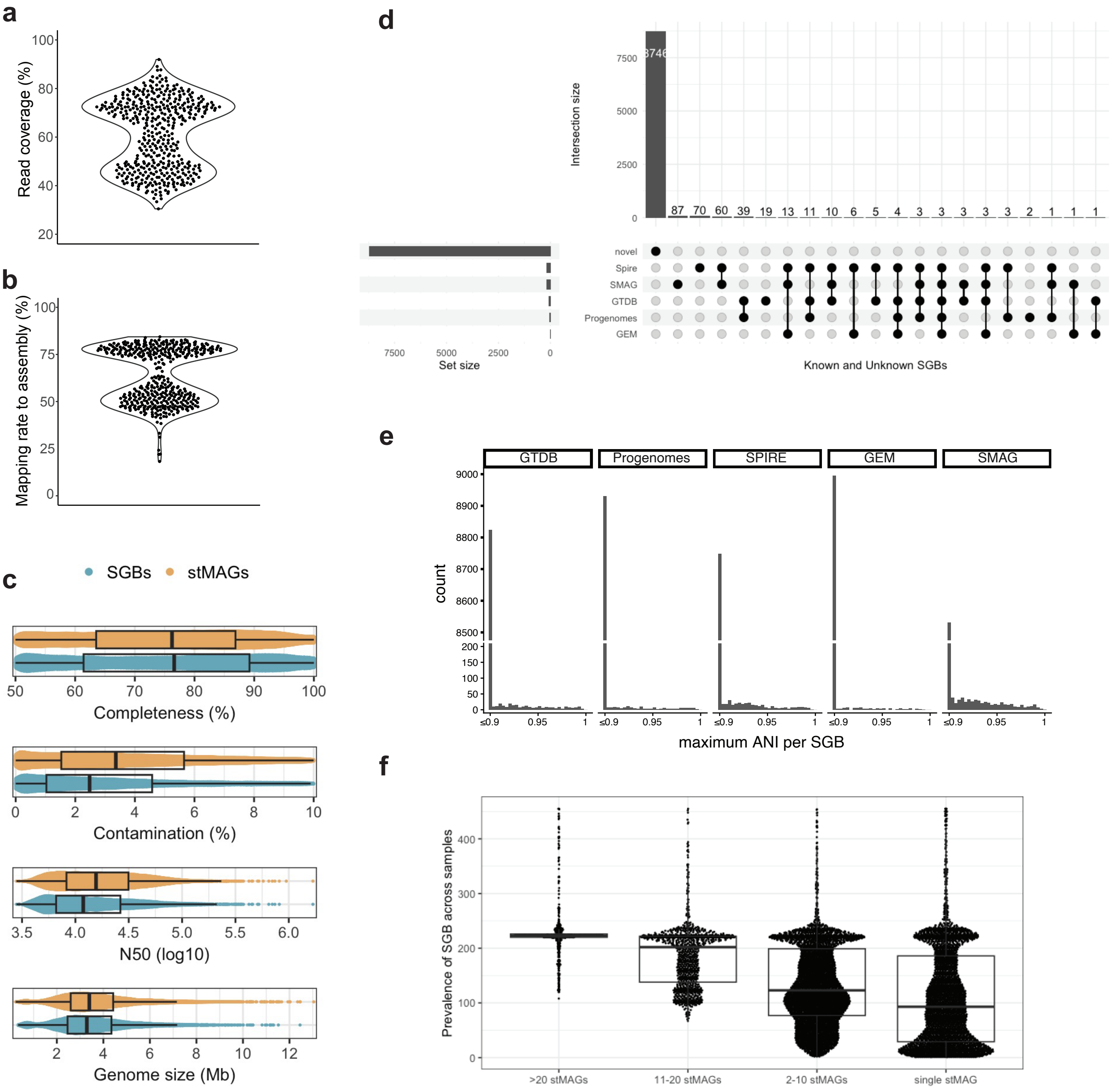

Supplementary Fig. 1 | Quality measures of agricultural metagenomic data and metagenome assembled genomes a, read coverage of 455 agricultural soil samples calculated using non-pareil. **b,** percentage of clean sequences mapping to the co-assembly for each of the 455 samples. **c,** distribution of genome quality metrics (completeness, contamination, N50 and genome size) of the 58,340 stMAGs and 9,090 SGBs. **d,** upset plot of the SGB catalogue matching to the different public genome catalogues. **e,** ANI distance with the closest reference genome per database for each SGB. **f,** SGB prevalence across the 455 samples separated by number of stMAGs in each SGB cluster. A SGB was considered present in a sample when relative abundance was >0.0001%.

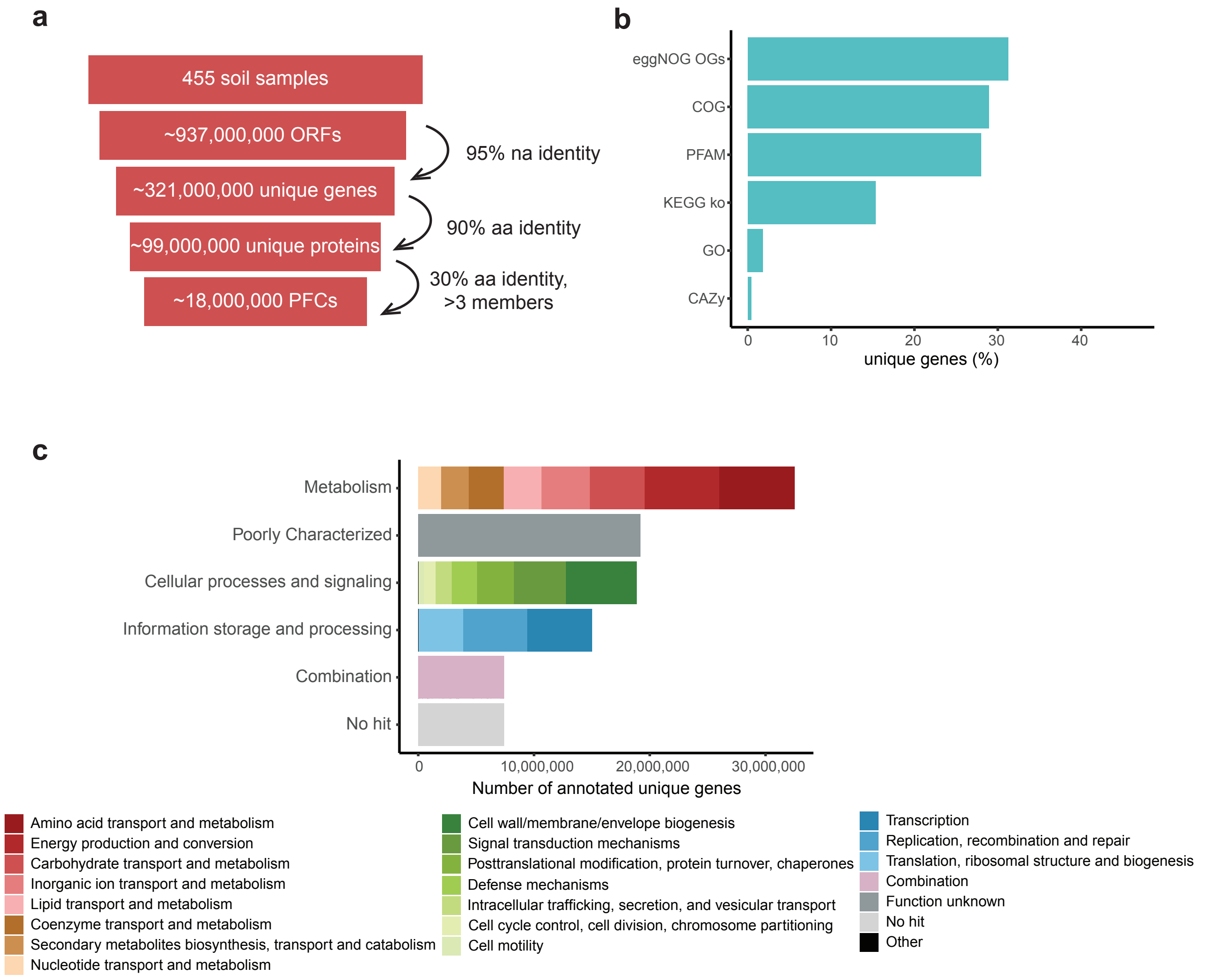

Supplementary Fig. 2 | Large mainly uncharacterized functional potential of soil microorganisms. a, more than 937k ORFs were predicted from 455 agricultural soil samples and further clustered into ~321k unique genes (>95% nucleic acid identity), ~99k unique proteins (90% amino acid identity) and ~18k protein family clusters. **b,** functional annotation of the unique genes catalog (95% amino acid identity) across different databases based on the EggNOG mapper. **c,** COG functional annotation of the unique gene catalogue (95% amino acid identity).

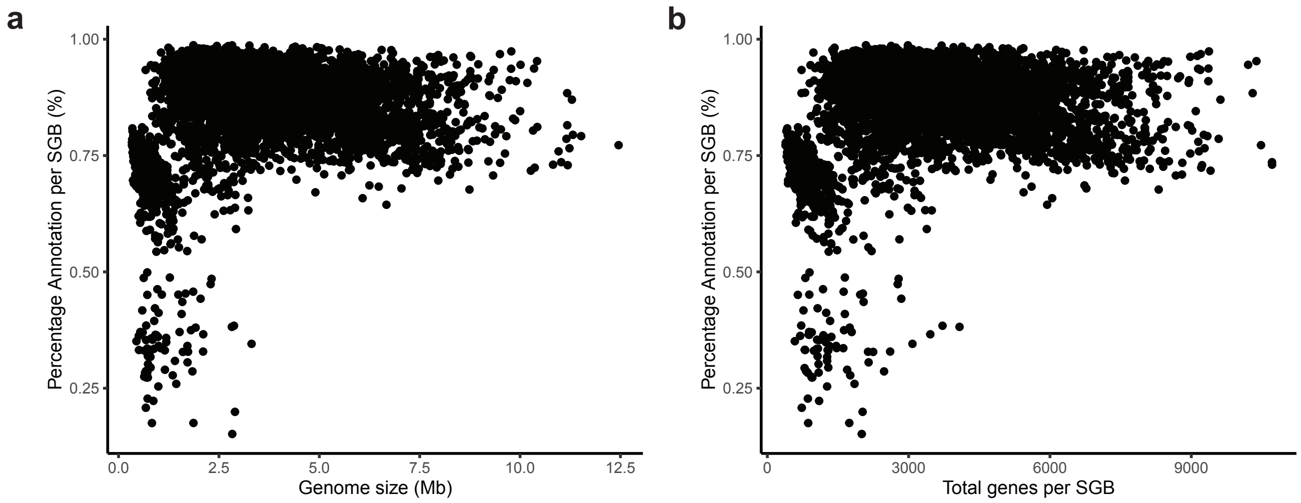

Supplementary Fig. 3 | **Percentage of gene annotation does not correlate to genome size. a,** percentage of gene annotation per SGB versus genome size (Mb). **b,** and total genes (ORFs) predicted per SGB.

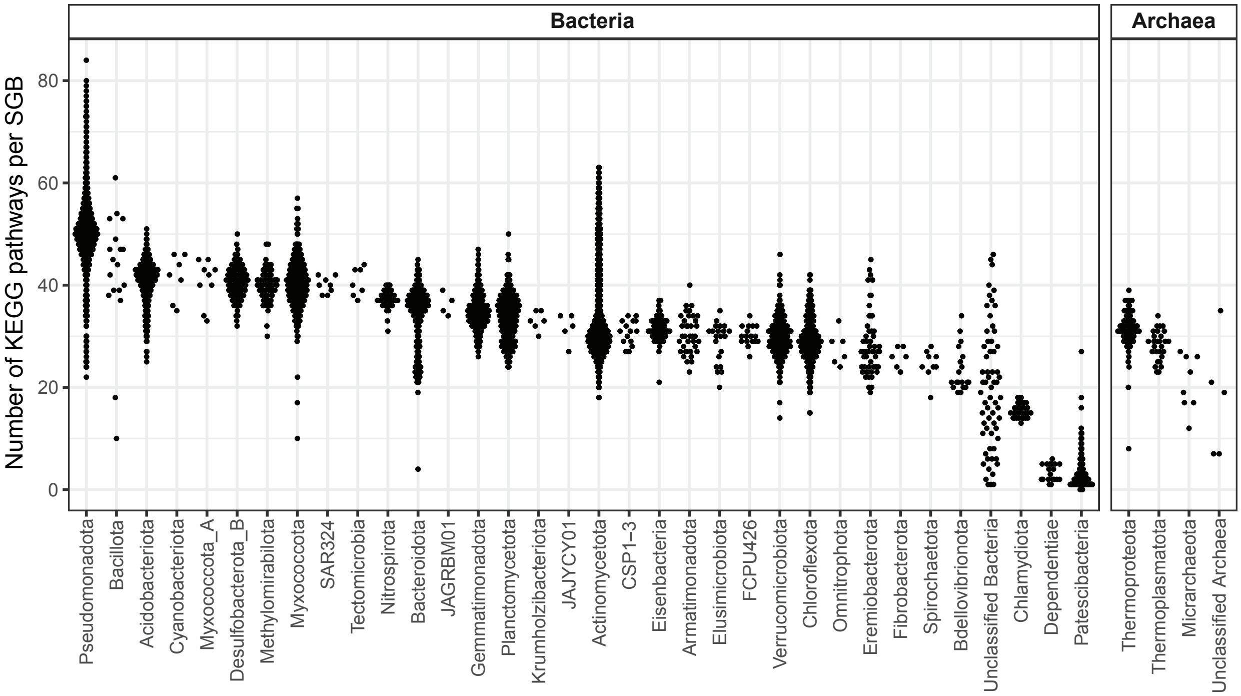

Supplementary Fig. 4 | Pathway annotation varies between agricultural soil microbial genomes in a phylogeny-dependent manner. The number of annotated KEGG modules among the 194 modules tested with MetapathPredict in each genome and ordered by phylum. Each dot describes one genome.

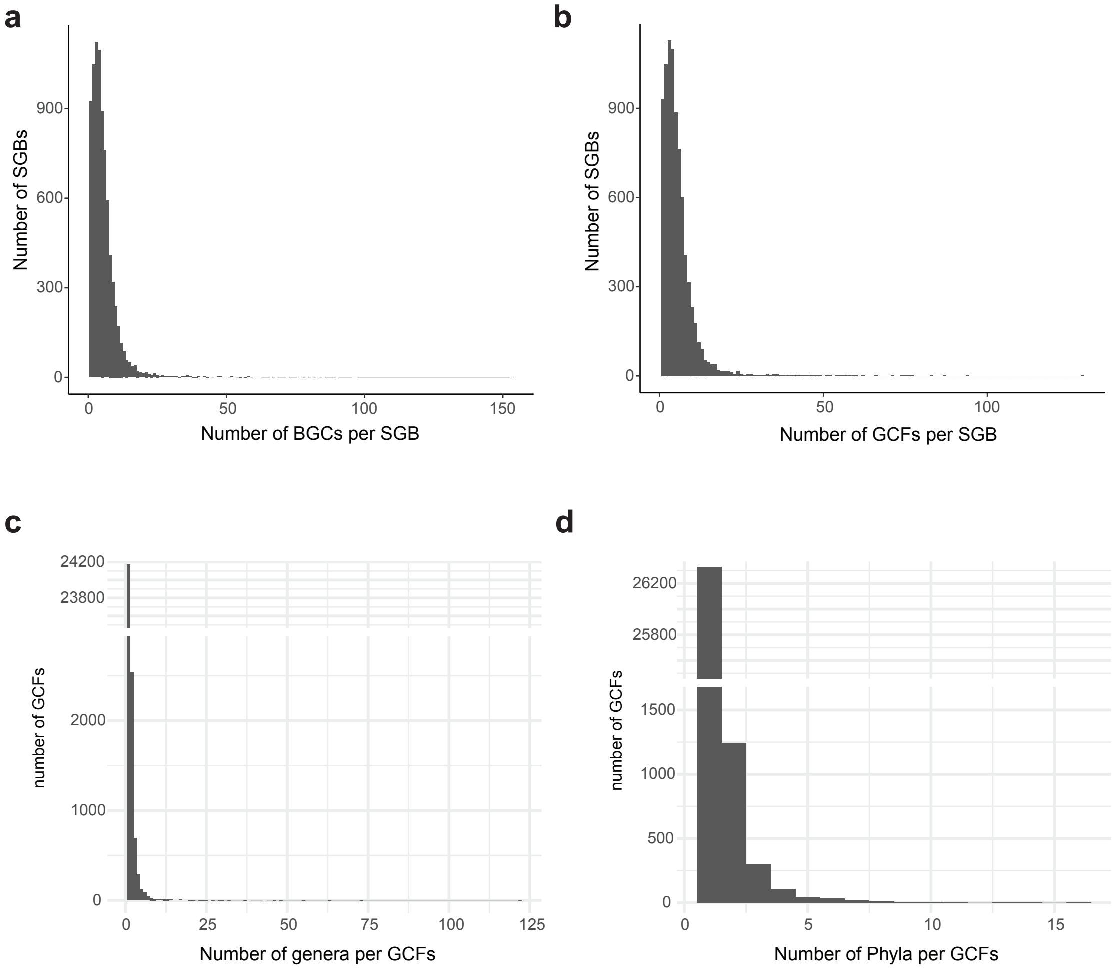

Supplementary Fig. 5 | **Distribution of BGCs and GCFs in soil prokayrotic genomes. a,** histogram of the number of BGCs per genome and **b,** the number of GCFs per genome. **c,** number of different genera that share the same GCF. **d,** number of different phyla that share the same GCF.

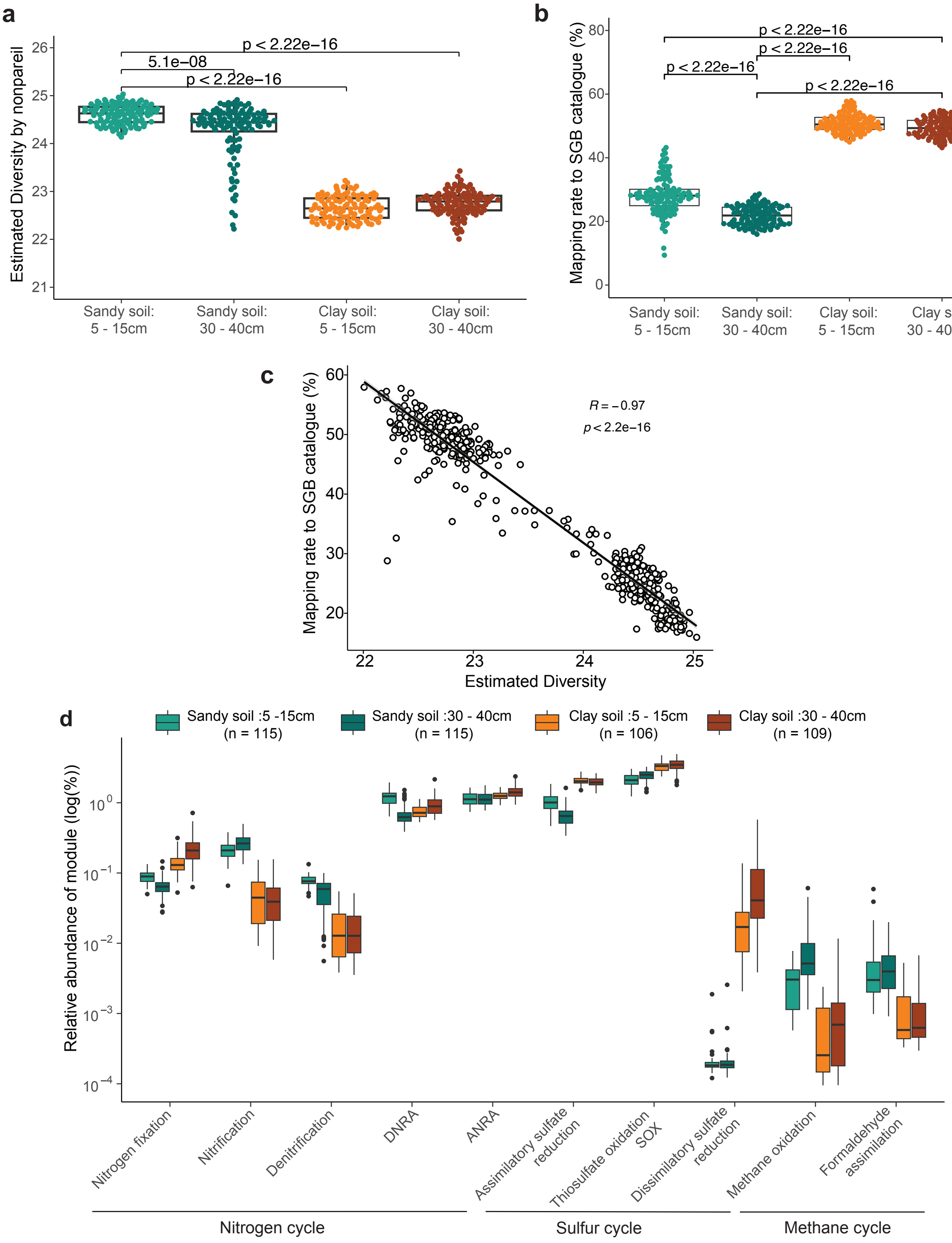

Supplementary Fig. 6 | Compositional and functional differences between soil types and depths. a, diversity estimated on read diversity - with non-pareil per field and soil depth. **b,** mapping rate of 445 agricultural soil samples based on CoverM **c,** Two-sided Spearman correlation of the estimated diversity vs the mapping rate to the SGB catalogue. **d,** relative abundance of all SGBs with a given KEGG module, for modules relevant to nitrogen, sulfur and methane cycle.

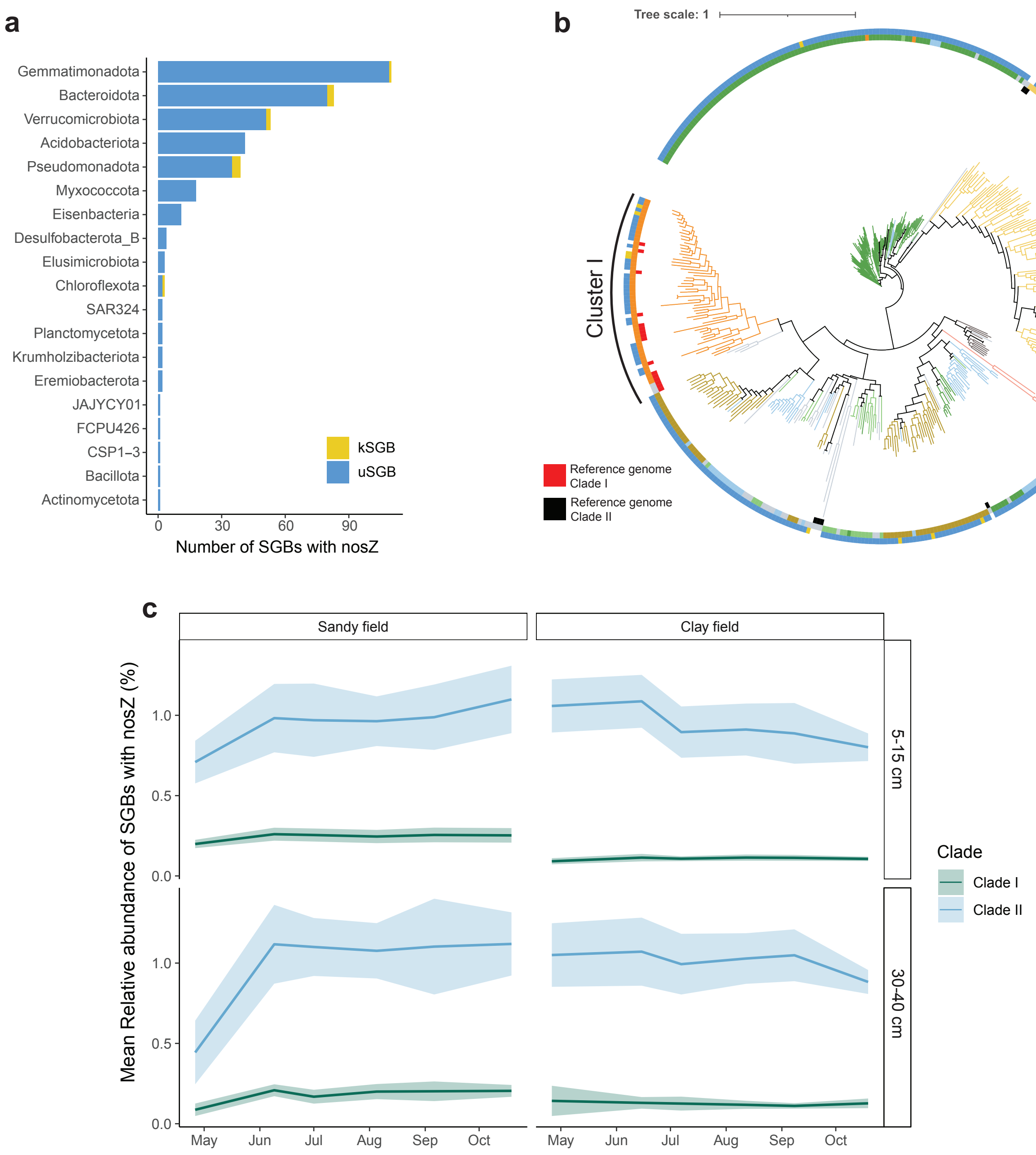

Supplementary Fig. 7 | nosZ gene distribution and abundance in soil microbiomes. a, SGBs that hold at least one nosZ gene plotted by phylum. **b,** phylogenetic tree of 378 nosZ genes detected in this study and 49 reference nosZ genes from NCBI. Reference genes are annotated according to clade. Taxonomy and novelty are also displayed in the outer rings. **c,** relative abundance of the genomes with a Clade I (green) or Clade II (blue) nosZ gene per timepoint, soil type and depth. The line represents the average relative abundance across samples, and the ribbon the standard deviation.

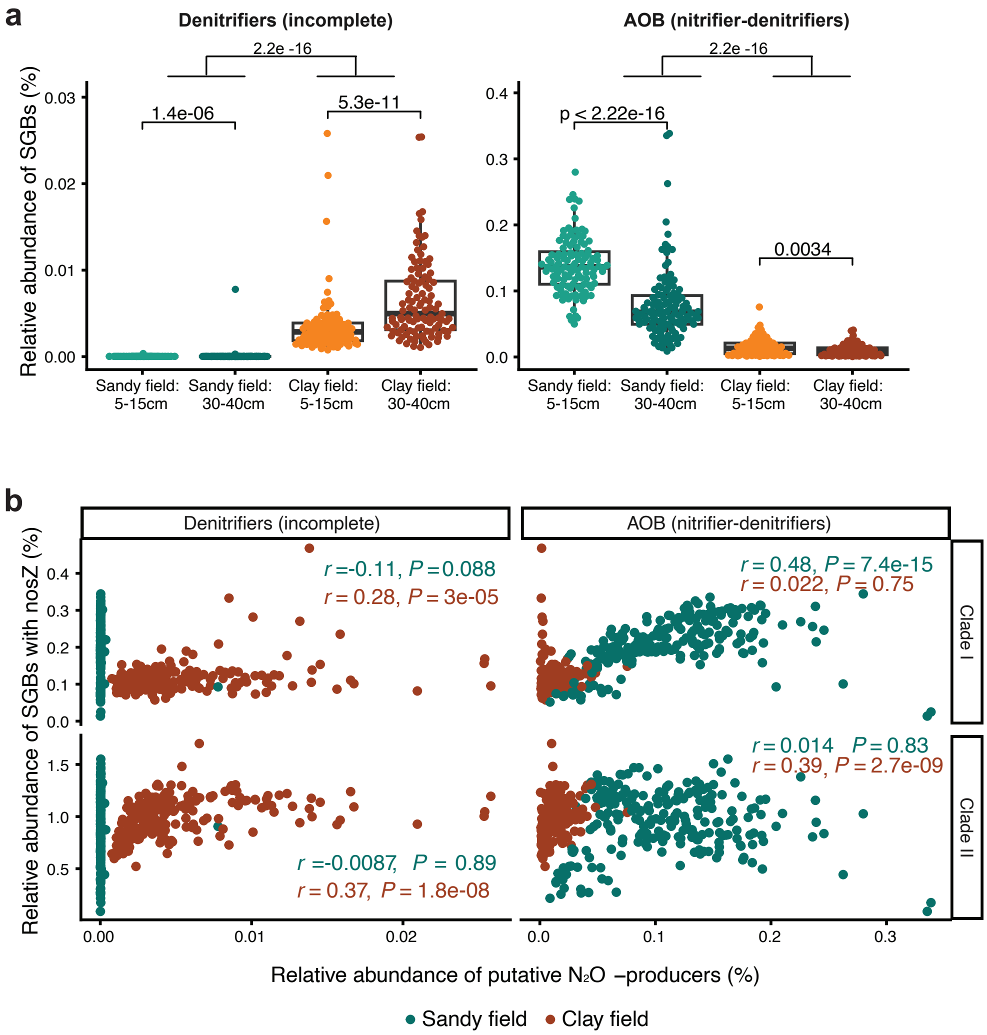

Supplementary Fig. 8 | Abundance of putative N_2O-producing species in soil microbiomes. a, Relative abundance of all putative N_2O-producing SGBs. Significant differences between soil types and depths were calculated with a two-sided Wilcoxon test. **b,** Relative abundance of SGBs with *nosZ* vs relative abundance of putative N_2O-producing SGBs split per *nosZ* clade and pathway of N_2O-production. The reported *r* and *P* values are based on a two-sided Pearson correlation.
